# Chromosome-level genome assembly of a critically endangered species *Leuciscus chuanchicus*

**DOI:** 10.1038/s41597-025-04787-2

**Published:** 2025-03-15

**Authors:** Qi Wang, Qi Zhou, Hongyan Liu, Jiongtang Li, Yanliang Jiang

**Affiliations:** Key Laboratory of Aquatic Genomics, Ministry of Agriculture and Rural Affairs, CAFS Key Laboratory of Aquatic Genomics, Chinese Academy of Fishery Sciences, Beijing, China

**Keywords:** Genome, Agricultural genetics

## Abstract

*Leuciscus chuanchicus,* a critically endangered cyprinid endemic in the Yellow River, represents an evolutionary significant lineage within Leuciscinae. However, conservation efforts for this species have been hindered by the lack of genetic and genomic resources. Here we reported a high-quality chromosome-level genome of *L. chuanchicus* by combining Illumina reads, PacBio HiFi long reads and Hi-C data. The assembled genome size was 1.16 Gb, with a contig N50 size of 31,116,631 bp and a scaffold N50 size of 43,855,677 bp. The resulting 130 scaffolds were further clustered and ordered into 25 chromosomes based on the Hi-C data, representing 97.84% of the assembled sequences. The genome contained 60.36% repetitive sequences and 35,014 noncoding RNAs. A total of 31,196 protein-coding genes were predicted, of which 28,323 (90.79%) were functionally annotated. The BUSCO and OMArk revealed 97.6% and 91.28% completion rates, respectively. This study assembled a high-quality genome of *L. chuanchicus*, and provided fundamental genomic resources for investigating the molecular mechanism and evolution of the Leuciscinae.

## Background & Summary

The genus *Leuciscus* (Cuvier, 1816) is the central group for understanding the phylogeny and systematics of Leuciscinae, and is apparently ancestral for many phylogenetic lineages within the subfamily^1,2^. More than 40 species are traditionally assigned to this genus, widely distributed throughout Eurasia^3^. Among them, *L. chuanchicus* is a medium-sized and omnivorous fish species, feeding mainly on aquatic insects, aquatic plants, and algae. It is an endemic species in the Yellow River, and is distributed only in the upstream of the Yellow River (Fishes of the Yellow river valley). Currently, the population of *L. chuanchicus* is small, and it is listed on the China Species Red List as a critically endangered (CR) species^4^. Despite its important status, the genetic and genomic resources for *L. chuanchicus* are scarce. It is a major challenge for biologists and ecologists to protect endangered species. In the recent era, genomics is becoming an increasingly important approach to conservation biology for understanding genetic diversity in threatened species. The genomic resources can provide detailed information about the present and past demographic parameters, phylogenetic issues, the molecular basis for integrating genetic and environmental methodologies to conservation biology, and for designing fast monitoring tools^5–7^. Unfortunately, due to limited budgets typically for the area of conservation biology, the price for generating a high-quality *de novo* assembly of most endangered species is still a challenge^8^. A solution to this problem is enabled by the advancements in long-read genome sequencing technologies combined with the high throughput chromosome conformation capture (Hi-C) technology, which can generate more contiguous assemblies containing scaffolds spanning the length of entire chromosome with inexpensive cost^9^.

The absence of genomic resources for *L. chuanchicus* has impeded both phylogenetic studies within Leuciscinae and evidence-based conservation planning. Our study filled this critical knowledge gap by generating the first chromosome-level genome assembly for this critically endangered fish species, and provided a fundamental genomic resources for investigating molecular evolution in Leuciscinae and suggesting genomic-based management strategies.

## Methods

### Ethics statement

This study was approved by the Laboratory Animal Ethics Committee of the Centre for Applied Aquatic Genomics at the Chinese Academy of Fishery Sciences. The sample collection process complied with the guidelines of Chinese Academy of Fishery Sciences.

### Sample collection

An adult *L. chuanchicus* was collected from Ningxia section of the Yellow River during the Yellow River fisheries resources and environment investigation on 2023. The collection of the sampled fish in this study was permitted by the Bureau of Fisheries and Fishery Administration, Ministry of Agriculture and Rural Affairs of the People’s Republic of China. Tissues from the *L. chuanchicus* were collected and immediately stored in liquid nitrogen until DNA or RNA isolation. High quality DNA was extracted using TIANamp Genomic DNA kit (Tiangen, Beijing, China). Total RNA was extracted using Animal Tissue Total RNA Extraction Kit (Tiangen) following the manufacturer’s instructions.

### Illumina sequencing and genome survey

**Fig. 1:**
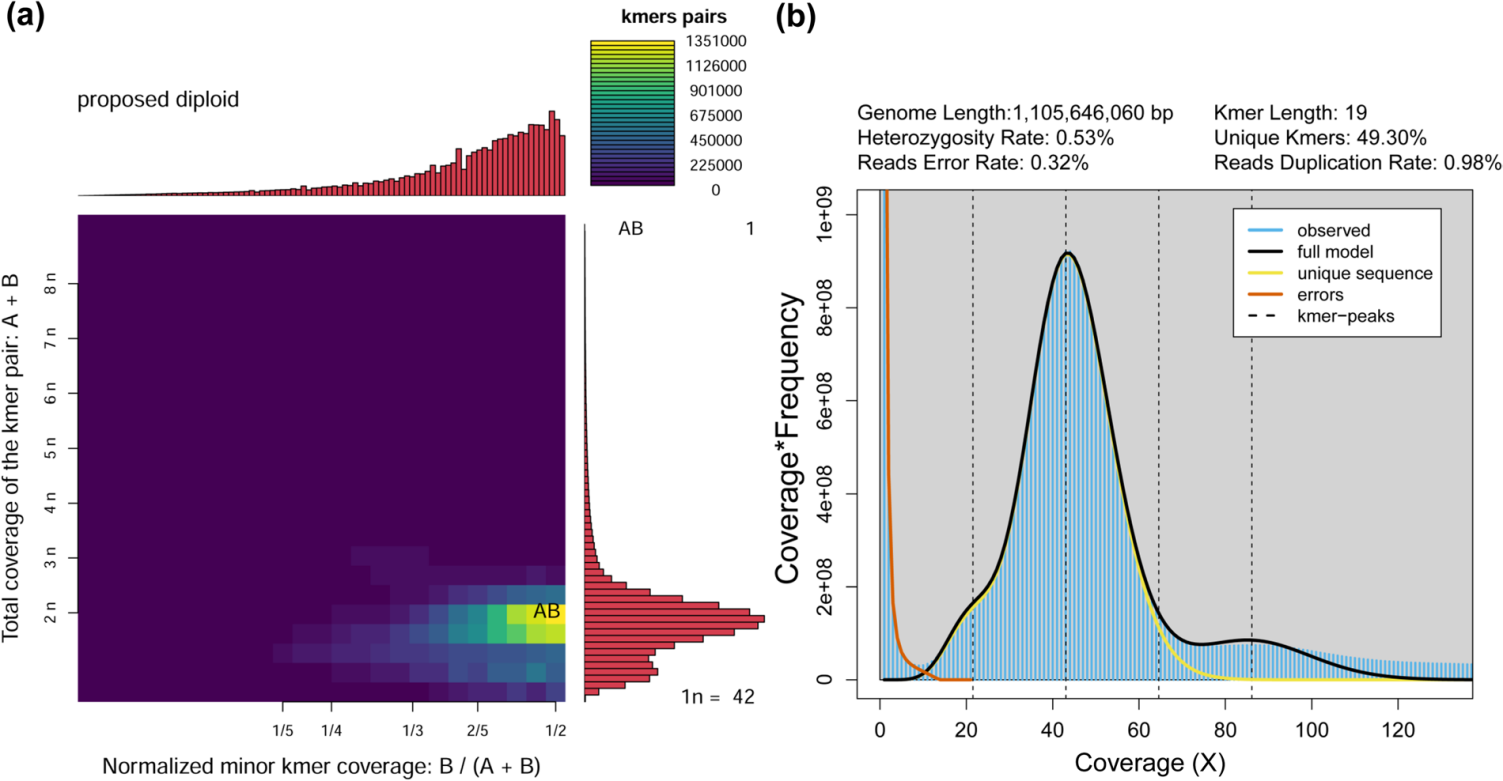
The estimated characteristics of *L. chuanchicus* genome. (**a**) Smudgeplot of ploidy estimation. (**b**) K-mer distribution used to estimate genome size.

Gnomic DNA was isolated from muscle tissues of a single fish. The quality of the DNA was assessed using agarose gel electrophoresis, Nanodrop, and Qubit Fluorometer. High-quality DNA was randomly sheared to 300–500 bp fragments, and a paired-end library was prepared following the manufacturer’s protocol. The library was sequenced on an Illumina NovaSeq 6000 platform using a paired-end 150 bp layout to enable genome survey and base-level correction. After removing low-quality, short reads, and adaptor sequences using Fastp v0.23.2^10^, a total of 385,955,792 clean reads were used for the genome survey (Table 1). The K-mer value was set at 19, and K-mer frequencies and K-mer pairs were calculated using KMC v3.2.2^11^. The generated smudgeplot confirmed the proposed diploid of *L. chuanchicus* (Fig. 1a). Further genome size and heterozygosity ratio was estimated by combining Jellyfish v2.3.0^12^ and GenomeScope v2.0^13^. The survey analysis results showed that the main peak was observed around a depth of 43 (Fig. 1b). The genome size was estimated to be 1.1 Gb, with a heterozygosity rate of 0.53% at K-mer = 19 (Fig. 1b).

**Table 1:** Statistics of the sequencing data.

Library type	Tissue	Insert size (bp)	Reads number	Total read bases (Gb)	Mean read length (bp)	N50 read length (bp)	Average coverage (X)
PacBio HiFi	Muscle	20,000	4,764,355	77.24	16212.62	16,299	69.86
Hi-C	Muscle	350	962,506,070	144.38	150	150	130.58
Illumina	Muscle	350	385,955,792	57.89	150	150	52.36
RNA-seq	Pooled	350	122,898,236	18.43	150	150	—

### PacBio HiFi sequencing and contig-level genome assembly

High-quality genomic DNA was sheared into fragments of approximately 15 Kb in size. After purification and size-selection, the qualified DNA fragments were used for SMRTbell library construction using a SMRTbell Express Template Prep Kit 2.0 (Pacific Biosciences, CA, USA). The library was sequenced on the PacBio Sequel Revio platform utilizing SMRT technology. The PacBio SMRT-Analysis package (https://www.pacb.com) was used for the quality control of the raw polymerase reads. Adaptor sequences, and the polymerase reads with short length or low-quality values were removed. HiFi reads were generated by SMRTLink software with parameters –min-passes = 3 –min-rq = 0.99. After removing low-quality sequences or contaminate sequences, a total of 4,764,355 high-precision long reads with an N50 value of 16,299 bp were obtained (Table 1). Then, the HiFi reads were used for *de novo* assembly by using Hifiasm v0.16.1 with defaulting parameters^14^. The resulting draft genome consists of 233 contigs, with a total length of 1,160,128,619 bp and N50 size of 31,116,631 bp (Table 2).

**Table 2:** Comparison of genome assemblies in three *Leuciscus* species.

Features	*Leuciscus waleckii* (2017)^53^	*Leuciscus waleckii* (2023)^20^	*Leuciscus chuanchicus*
Estimated genome size (bp)	896,000,000	1,125,030,000	**1,105,646,060**
Contig number	39,398	6,407	**233**
Total length (bp)	738,258,966	1,103,966,172	**1,160,128,619**
Contig N50 (bp)	37,373	1,515,867	**31,116,631**
Average contig length (bp)	18,738	172,603	**4,979,093**
Largest contig length (bp)	303,582	12,092,634	**50,979,897**
GC contents (%)	38.11	38.77	**38.87**
Scaffold number	4,888	4,250	**130**
Total length (bp)	752,538,629	1,105,256,174	**1,160,198,222**
Scaffold N50 (bp)	21,959,719	40,463,192	**43,855,677**
Average scaffold length (bp)	153,956	252,850	**8,924,602**
Largest scaffold length (bp)	37,168,685	71,368,982	**77,086,779**
GC contents (%)	37.39	38.69	**38.87%**
Number of chromosomes	24	25	**25**
Length of scaffolds anchored on chromosomes (bp)	556,215,515 (73.91%)	1,020,347,057 (92.32%)	**1,135,130,988 (97.84%)**
Gaps	34,510	2,582	**106**

Notes: The genome sequences and annotation data of *Leuciscus waleckii* (2017) were downloaded from NCBI database with accession number GCA_900092035.1, and the genome sequences and annotation data of *Leuciscus waleckii* (2023) were downloaded from NCBI database with accession number GCA_041200155.1.

### Hi-C sequencing and chromosomal-level genome assembly

**Fig. 2:**
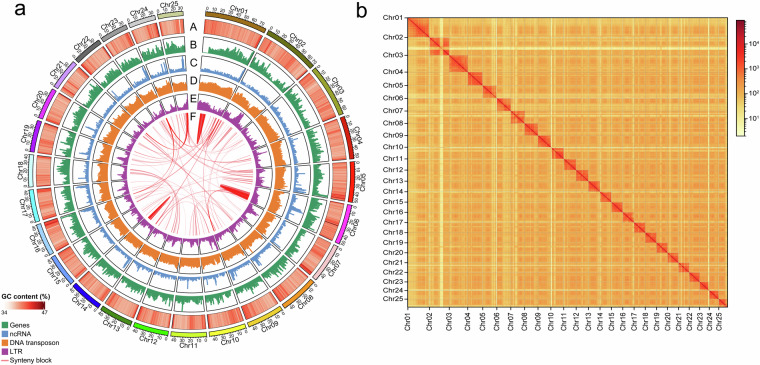
Genomic features and chromosomal interactions in the assembled *L. chuanchicus* genome showing by circos plot (**a**) and Hi-C interaction heatmap (**b**). Tracks in the circos plot from outer to inner layers depict the followings: “A” represents the GC content; “B” represents the gene density; “C” represents the ncRNA density; “D” represents the DNA transposon density; “E” represents the LTR retroelement density; “F” represents the synteny blocks.

To generate a chromosomal-level genome assembly, a Hi-C library was prepared using the genomic DNA isolated from the same *L. chuanchicus* fish sample, through a series process including crosslinking, cell lysis, chromatin digestion, biotin labelling, proximal chromatin DNA ligation, and DNA purification. The resulting library was sequenced on an Illumina NovaSeq 6000 platform. The adaptor sequences, low-quality reads, or reads with 3nt unidentified nucleotides were removed. The filtered Hi-C reads were aligned to the initial draft genome by HiCPro v2.11.4^15^, and only uniquely proper mapped paired-end reads were used for scaffolding by 3D-DNA v180922^16^. Juicebox v1.11.08^17^ was then used to order the scaffolds to obtain the final chromosomal-level assembly. The contact map was plotted using HiCExplorer v3.7.2^18^ (Fig. 2). The final genome of *L. chuanchicus* contained 25 chromosomes, covering 97.84% of the estimated nuclear genome (Table 2). Compared to the genome of *L. waleckii*^19,20^, a member of the same genus, the *L. chuanchicus* genome had a similar genome size, GC contents, and scaffold N50. However, the contig N50, average contig length, and largest contig length of our assembly were much longer, and the scaffold number and gap number was much less than that of *L. waleckii* (Table 2), indicating the high quality of our assembly of *L. chuanchicus* genome.

### Genome annotation

Repetitive elements in the *L. chuanchicus* genome were identified using a combination of *de novo* and homology-based methods. The employed tools included MITE-Hunter v1.0^21^, LTRharvest v1.6.2^22^, LTR Finder v1.07^23^, LTR retriever v2.9.0^24^, RepeatMasker v4.1.1^25^, and RepeatModeler v2.0.2a^26^. The results showed that the total length of repetitive elements was 667,325,904 bp, accounting for 60.3562% of the assembled genome. Among the repetitive elements, long terminal repeats (LTRs) and DNA transposons were the most abundant, accounting for 24.9193% and 22.5169% of genome, respectively (Table 3).

**Table 3:** Summary of repetitive elements in *L. chuanchicus* genome.

Type	Count	Length (bp)	Percentage(%)
DNA transposon	790,874	248,957,688	22.5169
LTR	658,557	275,519,088	24.9193
LINE	59,585	22,684,934	2.0517
Simple repeat	953	103,848	0.0094
SINE	525	62,412	0.0056
Unclassified	785,117	148,268,296	13.4101
Total	1,529,125	667,325,904	60.3562

For protein-coding gene prediction, total RNA was extracted from 9 tissues of *L. chuanchicus*, including skin, muscle, spleen, intestine, liver, kidney, heart, gill, and brain. Equal amounts of RNA from each tissue were pooled, and used to construct RNA sequencing library. The library was then sequenced on an Illumina NovaSeq 6000 platform. A comprehensive strategy combing *ab initio* prediction, protein-based homology searches, and RNA sequencing was employed to annotate the gene structure. AUGUSTUS v3.5.0^27^, SNAP v6.0^28^, GlimmerHMM v3.0.4^29^ and GeneMark-ET v4.32^30^ were used to *ab initio* predict gene structure in the repeat-masked genome. HISAT2 v2.2.1^31^ was used to align the filtered RNA-Seq reads to the genome sequences, and Cufflinks v2.2.1^32^ was used to assemble transcripts GeMoMa v1.9^33^ was then used to perform homology prediction and obtain exon-intron boundary information by comparing the transcript and genome sequences. A total of 31,196 protein-coding genes were successfully predicted in the genome, with an average gene length of 2,467.65 bp, an average CDS length of 1,767.79 bp, and an average exon number of 9.75 (Table 4). Comparing with other ten published fish genome including *Tiaroga cobitis*^34^, *Rhinichthys klamathensis goyatoka*^35^, *Pimephales promelas*^36^, *Meda fulgida*^37^, *L. waleckii*^20^, *Phoxinus phoxinus*^38^, *Megalobrama mblycephala*^39^, *Ctenopharyngodon idella*^40^, *Danio rerio*^41^, and *Oryzias latipes*^42^, showed that the number of protein-coding genes, average gene length, average CDS length, average exon length of *L. chuanchicus* genome were either similar or higher than that of most other fish species, indicating the high quality of the assembled transcriptome annotation. For functional annotation of these predicted genes, all protein-coding genes were aligned to EggNOG, Swiss-Prot, NR, KEGG, and GO database. Of all the predicted genes, 28,323 (90.79%) genes were successfully assigned with at least one functional annotation (Table 5).

**Table 4:** Comparison of the gene features of *L. chuanchicus* genome and other published fish genome.

Species	Number of protein-coding genes	Average gene length (bp)	Average CDS length (bp)	Average exon length (bp)	Average intron length (bp)	Average exons per gene
***L. chuanchicus***	**31,196**	**2,467.65**	**1,767.79**	**246.75**	**1,832.32**	**9.75**
*L. waleckii*	27,633	1,908.13	1,541.53	214.11	1,730.86	8.91
*P. phoxinus*	23,298	1,762.94	1,755.83	162.44	1,709.69	9.79
*P. promelas*	26,763	3,119.67	2,058.60	240.35	2,153.51	11.20
*M. fulgida*	38,742	1,608.79	1,588.36	223.26	936.93	7.21
*T. cobitis*	48,037	1,258.95	1,254.91	221.55	1,461.06	5.76
*C. idella*	25,255	3,971.82	2,266.95	284.80	2,559.28	12.22
*M. amblycephala*	30,620	3,473.54	2,136.64	264.73	2,474.60	10.89
*R. klamathensis goyatoka*	23,894	2,879.77	2,225.17	211.24	2,736.81	10.92
*D. rerio*	25,592	2,346.05	1,539.17	244.45	3,261.96	11.47
*O. latipes*	22,176	3,766.40	2,347.53	257.35	1,886.57	12.18

**Table 5:** Functional annotation of the predicted protein-coding genes.

Databases	Number	% of all predict genes
EggNOG	25,225	80.86
GO	17,260	55.33
KEGG	17,136	54.93
NR	28,312	90.76
Swiss-Prot	21,762	69.76
Total annotated	28,323	90.79

For non-coding RNA annotation, tRNAs were identified using tRNAscan-SE^43^, rRNAs were predicted using RNAmmer^44^, and ncRNA sequences were searched using INFERNAL v1.1.4^45^. Ultimately, 1,501 miRNA, 17,691 rRNAs, 1,521 snRNAs, and 14,301 rRNAs were identified in the genome, accounted for 0.011547%, 0.180541%, 0.019075%, and 0.091279%, respectively (Table 6).

**Table 6:** Statistics of the annotated non-coding RNAs.

Type		Number	Total length (bp)	% of genome
miRNA		1,501	133,963	0.011547
rRNA		17,691	2,094,513	0.180541
	5S	17,641	2,063,909	0.177904
	5.8S	2	305	0.000026
	18S	23	13,170	0.001135
	28S	25	17,129	0.001476
snRNA		1,521	221,298	0.019075
	snoRNA	305	42,694	0.003680
	splicing	1,204	177,946	0.015338
	Others	12	658	0.000057
tRNA		14,301	1,058,954	0.091279

## Data Records

All raw sequencing data including Illumina sequencing data, PacBio HiFi data, Hi-C sequencing data, and transcriptome data have been deposited in the NCBI Sequence Read Archive (SRA) under the accession number SRR29666729^46^, SRR29666730^47^, SRR29666728^48^, and SRR29666727^49^, respectively. The genome assembly and annotations have been deposited to ENA database under the accession number ERP169078^50^.

## Technical Validation

The quality and completeness of the genome assembly were further evaluated by using BUSCO^51^ with the actinopterygii_odb10 reference gene set. The final genome assembly showed a BUSCO completeness of 97.6%, consisting of 3,479 (95.6%) single-copy BUSCOs, 72 (2.0%) duplicated BUSCOs, 26 (0.7%) fragmented BUSCOs, and 63 (1.7%) missing BUSCOs (Table 7). The accuracy of the draft assembly was also assessed by mapping Illumina paired-end reads onto the assembled genome sequences. Of total reads, 99.63% were successfully mapped, 93.7% of which were properly paired-end mapped reads, achieving a good genome coverage of 99.97% (Table 8). Further proteome quality assessment was performed using BUSCO and OMArk. The BUSCO assessment showed a completeness of 93% (Table 7), while the OMArk assessment revealed that 91.28% of 16,357 Otophysi hierarchical orthologous groups were complete (Table 9). The high completeness of BUSCOs, high nucleotide-level accuracy, high completeness of OMArks, together with considerable continuity of contig sizes collectively suggest the high quality of genome assembly and annotation of *L. chuanchicus* produced in this study.

**Table 7:** BUSCO assessment statistics of the genome assembly.

Type	Genome	Transcriptome	Proteome
Complete BUSCOs	3,551 (97.6%)	3,409 (93.6%)	3,385 (93.0%)
Complete and single-copy BUSCOs	3,479 (95.6%)	3,321 (91.2%)	3,307 (90.9%)
Complete and duplicated BUSCOs	72 (2.0%)	88 (2.4%)	78 (2.1%)
Fragmented BUSCOs	26 (0.7%)	48 (1.3%)	47 (1.3%)
Missing BUSCOs	63 (1.7%)	183 (5.1%)	208 (5.7%)
Total BUSCOs	3,640	3,640	3,640

**Table 8:** Summary of paired-end reads mapping to the assembled *L. chuanchicus* genome.

Type	Value
Mapping ratio (%)	99.63
Properly paired (%)	93.70
Singletons (%)	0.21
Coverage > = 1x (%)	99.97
Coverage > = 10x (%)	99.51
Mismatch (%)	0.0046

**Table 9:** OMArk assessment statistics of proteome quality.

	Types	Number	Percentage
Completeness assessment (Ancestral clade used: Otophysi)	Total conserved HOGs	16357	—
	Completed	14931	91.28%
	single	14306	87.46%
	duplicated	625	3.82%
	expected	96	0.59%
	unexpected	529	3.23%
Whole proteome assessment	Number of proteins in the whole proteome	31196	
	Consistent lineage placement	24556	78.72%
	partial hits	3915	12.55%
	fragmented	661	2.12%
	Inconsistent lineage placement	2402	7.70%
	partial hits	1673	5.36%
	fragmented	163	0.52%
	Contamination	0	0.00%
	Unknown	4238	13.59%

## Data Availability

All commands and pipeline used in data processing were executed according to the manuals/protocols of the software. No specific code has been developed in this study. The main analysis scripts used in this study has been deposited in FigShare repository^52^.
